# Who Uses Smoking Cessation Apps? A Feasibility Study Across Three Countries via Smartphones

**DOI:** 10.2196/mhealth.2841

**Published:** 2014-02-06

**Authors:** Nasser F BinDhim, Kevin McGeechan, Lyndal Trevena

**Affiliations:** ^1^Sydney Medical SchoolDepartment of Public HealthUniversity of SydneySydneyAustralia; ^2^Public Health and Health Informatics schoolCollege of Health SciencesSaudi Electronic UniversityRiyadhSaudi Arabia

**Keywords:** smartphone, handheld computers, health promotion, tobacco and smoking, global health, prevention, apps, health Informatics, public health

## Abstract

**Background:**

Smartphone use is growing worldwide. While hundreds of smoking cessation apps are currently available in the app stores, there is no information about who uses them. Smartphones also offer potential as a research tool, but this has not previously been explored.

**Objective:**

This study aims to measure and compare the uptake of a smoking cessation app over one year in Australia, the United Kingdom, and the United States. It also assesses the feasibility of conducting research via an app, describing respondents’ characteristics (demographics, smoking status, and other health related app use), and examining differences across countries.

**Methods:**

This is a cross-sectional exploratory study of adults 18 years and older, passively recruited over one year in 2012, who downloaded this study app (Quit Advisor) via the two largest app stores (Apple and Android).

**Results:**

The total number of app downloads after one year was 1751, 72.98% (1278/1751) of them were Apple operation system users. Of these 1751 participants, 47.68% (835/1751) were from the United States, 29.18% (511/1751) were from the United Kingdom, and 16.68% (292/1751) were from Australia. There were 602 participants, 36.75% (602/1638) that completed a questionnaire within the app. Of these 602 participants, 58.8% (354/602) were female and the mean age was 32 years. There were no significant differences between countries in terms of age, operation system used, number of quitting attempts, and language spoken at home. However, there were significant differences between countries in terms of gender and stage of change. There were 77.2% (465/602) of the respondents that were ready to quit in the next 30 days and the majority of these had never sought professional help (eg, “Quitline”). More than half had downloaded smoking cessation apps in the past and of these, three-quarters had made quitting attempts (lasted at least 24 hours) using an app before. Respondents who had attempted to quit three times or more in the previous year were more likely to have tried smoking cessation apps (OR 3.3, 95% CI 2.1-5.2). There were 50.2% (302/602) of the respondents that had used other health related apps before. Of these, 89.4% (270/302) were using health related apps at least once a week, but 77.5% (234/302) never checked the credibility of the health app publishers before downloading.

**Conclusions:**

A smartphone app was able to reach smokers across three countries that were not seeking professional help, but were ready to quit within the next 30 days. Respondents were relatively young and almost demographically similar across all three countries. They also frequently used other health related apps, mostly without checking the credibility of their publishers.

## Introduction

### Smartphones and Apps

A smartphone is a mobile phone handset with advanced hardware and software capabilities that enable it to perform complex functions [1]. Consumers can utilize the advanced functionalities of smartphones and download applications (apps) from app stores. These apps are as capable as those that can be run on laptops, and can replace them in most functions, such as Web browsing, document processing, video and music playing, and task management. The smartphones’ portability makes apps available to consumers anytime and anywhere, which saves time and offers more privacy and anonymity. Such proximity to the consumer gives the smartphone great potential as a health promotion tool.

By the end of 2012, smartphone ownership accounted for 76% of all mobile phone handsets in Australia [2], 39% in the United Kingdom [3], and 55.5% in the United States [4]. The Android operating system (OS) and Apple OS devices dominate these uptake rates [2,4,5]. However, Android smartphone ownership is almost double that of Apple OS devices in both the United Kingdom and the United States [4,5].

App stores are not ordinary Web-based stores. They attract millions of users who seek apps for their smartphones. The largest are the Google Play (previously known as the Android Market) and the Apple App Store. In 2009, after nine months in business, the Apple App Store had uploaded one billion apps to its users [6]. In 2012, Apple users had downloaded 40 billion apps, up from 15 billion in 2011 [7,8]. Android users downloaded 25 billion apps in 2012, up from 10 billion in 2011 [9,10]. The app stores also allow the app owner to select which countries they want their app released in, allowing only users in those countries to see the app and download it. This offers an opportunity for researchers to explore the efficiency or effectiveness of health apps in a selected country without contaminating the results from users from other areas of the world. However, the accuracy of such function has never been tested before.

### Smartphones and Smoking Cessation Programs Delivery

The most widely used self-help smoking cessation program delivery method is printed documents [11,12]. However, printed self-help materials have disadvantages such as printing costs, limited distribution, lack of interactivity, and are limited in their ability to tailor for individual needs [11,12]. By contrast, computerized smoking cessation interventions eliminate printing costs, make updating easier, and can include interactivity and tailored intervention features [13,14]. Updating them, however, requires the user to download updates from the Internet, CD, or other computer media. The only advantage of computer-based interventions over Internet-based interventions is that computers do not require an Internet connection. Internet-based smoking cessation interventions have the advantages of anonymous online chat groups, bulletin discussion boards, and email, where health consumers can discuss sensitive personal health issues more comfortably than they can face-to-face in self-help groups [15].

The smartphone apps bring together the advantages of computer-based and Internet-based smoking cessation interventions. They also overcome their limitations. When there is no Internet connection, the user can still benefit from the computational interactive function and static information in the apps. The apps can host all kinds of multimedia, such as static and interactive rich-text, pictures, audio and video, and get more content when there is an Internet connection, without any user effort. Smartphone apps can also aid interactive self-monitoring by letting users add data about their health in various ways, including question and answer forms, text writing, and audio or video recordings. The apps can process, organize, and graph this self-monitoring data to help users understand their progress. Using this data can help the users in every step of their quitting journey, providing text information about quitting, letting them see how many days they are nicotine-free, providing diaries for their quitting attempts and craving triggers, and sending them reminders and motivational messages.

Dozens of smoking cessation apps are in smartphone stores, some with exaggerated claims of effectiveness [16]. No studies have yet assessed their uptake or their feasibility to be used as an intervention as well as a research tool. Protocols of randomized controlled trials to examine the effectiveness of smartphone apps to assist smokers in their quitting are emerging. A recent published protocol will examine a health care professional consultation plus smartphone app versus a standard counseling in participants recruited at primary care centers with 6 months follow up [17]. The study has not mentioned the OS that will be utilized [17]. Furthermore, another trial protocol by the authors will examine the effectiveness of an interactive smoking cessation self-help app versus standard smoking cessation information (including information about smoking consequences, quitting options, etc) that will be provided via a smartphone app [18]. The participants will be recruited directly from the Apple app store via one app that after identifying eligibility will randomize the participants to one of the subapps and follow their quitting attempt at 4 time points (10 days, 1 month, 3 months, and 6 months) [18]. Both studies are examining various ways to utilize smartphone apps in smoking cessation assistance and via different recruitment methods [17,18]. Pending the results of these two studies, more information is needed about the actual users of smoking cessation apps to help in customizing future interventions to target these users and to assess the feasibility of using smartphones to collect data. In addition, more information about smoking cessation apps’ uptake and users’ demographics in different OSs will help future research decide which systems to target.

In 2011, a study analyzed the content of 47 smoking cessation apps in the Apple App Store and found most of them were not evidence-based, particularly the most popular ones. Very few provided information about nicotine replacement or other effective quitting methods [16]. In addition, harmful prosmoking apps have found their way onto the market. In a recent paper, we identified 107 prosmoking apps; 42 were from the Android Market and downloaded by an average of 11 million users [1]. Some of those apps claim they can help people quit smoking [1]. Therefore, efforts are needed to bring evidence-based smoking cessation materials into this new medium.

Very little is known about app users or the ability of app stores to reach smokers. Thus, before examining the effectiveness of smoking cessation apps, we must know as much as we can about their potential users.

This exploratory study examined the feasibility of a free smoking cessation smartphone app developed for this study and published in the Apple App Store and Android Market to reach smokers. The study was designed to: (1) measure and compare the uptake of a smoking cessation app over one year in Australia, the United Kingdom, and the United States; (2) assess the feasibility of the smartphone as a research tool describing respondents’ characteristics (demographics, smoking status, and other health related app use); (3) examine differences between respondents from each country; and (4) investigate the association between smoking status and smokers’ use of smoking cessation apps.

## Methods

### Design and Recruitment

**Figure 1:**
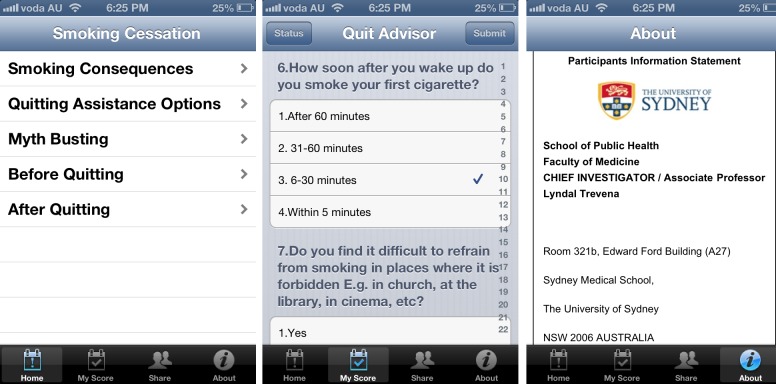
Screenshots of the study app.

This is a descriptive, cross-sectional, exploratory study of a convenience sample of adults 18 years and older, passively recruited over one year, who downloaded Quit Advisor (QA) via the app stores. As part of this study, we developed a free smartphone smoking cessation app (QA) and released it in the Apple and Android stores in Australia, the United States, and the United Kingdom in April 2012. Consumers in those stores and countries downloaded the app after viewing the study information and consent. The app page summarized consent information before download and we also included it in the terms of use agreement. We also included the consent form and participant information sheet inside the app “About” section so users can get back to it easily (Figure 1 shows a screenshot of the study app).

### App Design and Data Collection

QA is a smartphone app developed specifically for this study. It contains two major parts: (1) evidence-based information about smoking cessation, and (2) a questionnaire. The questionnaire collected data about demographics, smoking behavior, and nicotine dependency using the Fagerström scale [19], stage of change [20,21], previous use of smoking cessation apps, and general use of health related apps. When the user opened the app for the first time, it extracted the unique device identifier, encrypted it, and registered the user in the study’s database. This allowed anonymous data collection, eliminated the need for registration, and prevented duplication, as each device has only one chance of being in our records, even if the user reinstalls the app or the OS. The app’s first section, “Home” (Figure 1), contained information such as health effects of smoking and quitting options collected from recent research and health reports. The average level of the information on the Flesch-Kincaid readability test was 8th grade (age 14). Any time the user opened a category, the app sent the time and date of each view to the Web-based database as soon as an Internet connection was available. At the end of each category there was: (1) a reference list, and (2) an optional Likert-scale question to rate the motivational effect of the provided information in each category. In the second section, “My Score” (Figure 1), users completed the questionnaire. After submitting it, they received feedback about their nicotine dependency. Answers automatically went to the study’s database. Participants could only submit the questionnaire once, even if they deleted and reinstalled the app. Participants could not submit the questionnaire without completing all questions. The app was pretested by a group of users in various situations to assure the accuracy of the transferred data, and to test the other app functions such as country detection. In addition, to minimize the possibility of some users installing the app on more than one device, we have implemented a server-side Internet Protocol (IP) monitoring that can identify the users that use different devices connected to the same Internet network at similar times. Although this function was pretested successfully, no cases were identified during the data collection. Finally, because the app stores accuracy of limiting the app to specific countries is unknown, we have implemented a location identification function to know the users country the first time they open the app.

### Analysis

Descriptive analysis was used to assess the uptake of the app and the characteristics of the users’ ratings of the information categories. Bivariate analysis (chi-square and one way analysis of variance) helped examine the differences between countries in terms of user characteristics. Logistic regression controlling for demographics (age, gender, country, and education level) was used to investigate variables related to prior use of smoking cessation apps and the effect of app type on quit attempts.

## Results

### Uptake

**Figure 2:**
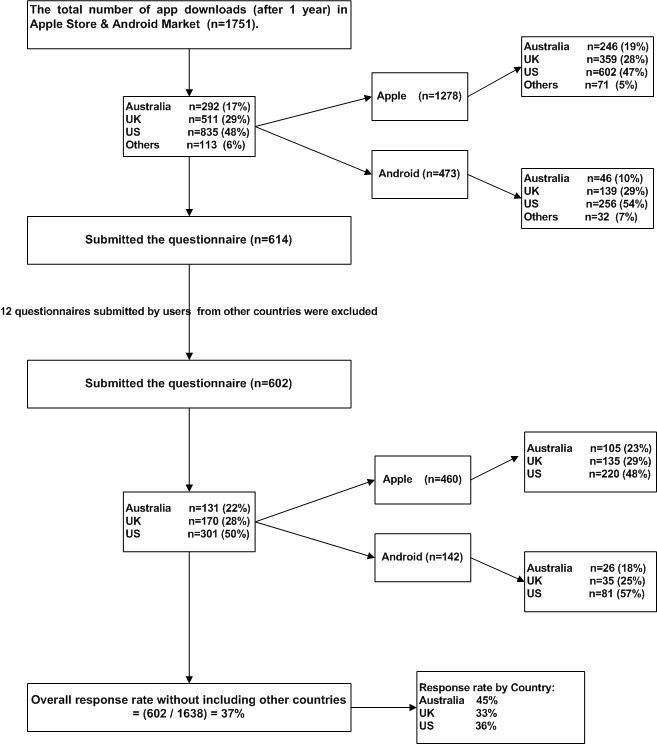
Flowchart of the recruitment process. The percentages were rounded up to the nearest whole.

Figure 2 shows, in one year, 1751 users downloaded the app from both stores, 72.98% (1278/1751) from the Apple Store and 27.01% (473/1751) from the Android Market. Of those 1751, 47.68% (835/1751) were from the United States, 29.18% (511/1751) from the United Kingdom, 16.68% (292/1751) from Australia, and 6.45% (113/1751) from other countries. The lowest Android download rate was in Australia at 9.7% (46/473), compared with 19.25% (246/1278) for Apple OS. After excluding users who submitted the questionnaire from other countries, 602 had submitted it, with an overall response rate of 36.75% (602/1638). The highest response rate was 44.8% (131/292), from Australia.

### Participants' Characteristics

Of those 602 who submitted the questionnaire, 50.0% (301/602) were from the United States, 28.2% (170/602) from the United Kingdom, and 21.8% (131/602) from Australia. The majority of participants 76.4% (460/602) were using Apple devices. The participants’ mean age was 32 and the median 31 years (18-67). Female participants outnumbered males by 17.6% (106/602). The most common level of education reported was “High School.” A chi-squared (χ^2^) test showed a significant education-level difference between countries (Table 1). Countries differed significantly in gender distribution, with women only outnumbering men in the United States. After assuring the homogeneity of variance a one-way analysis of variance revealed no significant differences between countries in terms of age–*F*_2,601_= 2.6, *P*=.07. Furthermore, there were no significant differences between countries in term of language spoken at home and OS used.

### Smoking Status

The Fagerström scale puts 44.5% (268/602) of the participants at a low or very low nicotine dependency, and 55.5% (334/602) at a medium to very high nicotine dependency (Table 1). After assuring the homogeneity of variance, a one-way analysis of variance revealed significant national differences in nicotine dependency scores, *F*_2,601_=4.4, *P*=.01. Post hoc comparisons using Tukey’s HSD test indicated that the mean score for Australia (mean 5.4, SD=2.4) was significantly higher than that of the United States (mean 4.6, SD=2.4). UK participants (mean 4.7, SD=2.7) did not differ significantly from either Australian or US participants.

Most participants 77.2% (465/602) were willing to quit in the next 30 days (preparation stage of change), and most of them 67.6% (407/602) had attempted to quit at least once in the previous year. A quitting attempt was defined as one that had lasted at least 24 hours. There was a significant difference between countries in terms of willingness to quit, but none in terms of previous quitting attempts.

There were 88.7% (401/452) of participants that had not contacted the “Quitline” services in their countries in the last year, with no significant variation between countries χ^2^=0.7, *P*=.71. Moreover, 71.7% (324/452) of participants had not contacted their health care professionals regarding quitting in the last year. However, here there was a significant variation χ^2^=7.9, *P*=.019, with 38% (36/93) of Australian participants contacting health professionals compared to 30.0% (36/120) in the United Kingdom, and 23.4% (56/239) in the United States.

There were 54.5% (328/602) of the participants that had used smoking cessation apps in the past, and the majority 75.6% (248/328) had made a quitting attempt that lasted at least 24 hours using an app. There were no significant differences between countries in terms of using smoking cessation apps in the past and making quitting attempts using those apps. There were 33.0% (82/248) of those who made quitting attempts using an app that had lasted more than one week abstaining from smoking.

Three or more quit attempts in the past year were associated with previous smoking cessation app use (OR 3.3, 95% CI 2.1-5.2). There was a suggestion of a dose-response relationship between the number of quit attempts and smoking cessation app use with two or more attempts also being associated (OR 2.9, 95% CI 11.8-4.7). In addition, difficulty to refrain from smoking in banned areas was associated with the likelihood of the participants trying smoking cessation apps (OR 1.5, 95% CI 1.0-2.1).

“Smoking counter” apps were the most frequently used, followed by “motivation” apps (Table 2). We have investigated if the use of any of these app types was associated with quitting attempts, controlling for age, gender, country, and education level. To do this, we asked users if they have used smoking cessation apps previously, what type of apps they have used, and if they tried to quit using these apps. We found an association between “smoking counter” apps and participants quitting attempts in the past (OR 2.2, 95% CI 1.2-3.6).

**Table 1:** Participants’ characteristics by country (n=602).

			Country				Total	χ^2^ (*P*)
Characteristics			AU n (%)	UK n (%)		US n (%)	n (%)	
Age, mean (SD) (years)			33.2 (9.4)	30.8 (9.9)		32.6 (10.2)	32.2 (9.9)	-
**Sex**								30.4 (.001)
	Female		65 (49.6)	79 (46.5)		210 (69.8)	354 (58.8)	
	Male		66 (50.4)	91 (53.5)		91 (30.2)	248 (41.2)	
**Education**								28.7 (.001)
	High school		73 (55.7)		67 (39.4)	161 (53.5)	301 (50.0)	
	Diploma/Associate		12 (9.2)		33 (19.4)	57 (18.9)	102 (16.9)	
	Graduate (Bachelor)		21 (16)		25 (14)	32 (10)	78 (13)	
	Master Degree or higher		5 (3)		5 (2)	19 (6)	29 (4)	
	Others		20 (15)		40 (23)	32 (10)	92 (15)	
**Language spoken at home**								5.5 (.64)
	English		117 (89.3)		157 (92.4)	287 (95.3)	561 (93.2)	
	Others (14 Languages)		14 (10)		13 (7)	14 (4)	41 (6)	
**OS**								3.7 (.15)
	Apple		105 (80.2)		135 (79.4)	220 (73.1)	460 (76.4)	
	Android		26 (19.8)		35 (20.6)	81 (26.9)	142 (23.6)	
**Nicotine dependency (Fagerström)**								20.9 (.009)
	Very low (0-2)		21 (16.0)		39 (22.9)	64 (21.3)	124 (20.6)	
	Low (3-4)		23 (17.6)		39 (22.9)	82 (27.2)	144 (23.9)	
	Medium (5)		17 (13)		28 (16)	39 (13)	84 (14)	
	High (6-7)		43 (32.8)		31 (18.2)	82 (27.2)	156 (25.9)	
	Very high (8-10)		27 (20)		33 (19)	34 (11)	94 (15)	
**Stage of change**								11.2 (.02)
	Next 30 days (preparation)		93 (71.0)		125 (73.5)	247 (82.1)	465 (77.2)	
	Next 6 months (contemplation)		28 (21.4)		31 (18.2)	45 (15.0)	104 (17.3)	
	Not thinking of quitting (precontemplation)		10 (7)		14 (8)	9 (3)	33 (5)	
**Number of quitting attempts last year**								4.9 (.55)
		1 time	35 (26.7)		33 (19.4)	56 (18.6)	124 (20.6)	
		2 times	21 (16.0)		36 (21.2)	66 (21.9)	123 (20.4)	
		3 or more	32 (24.4)		46 (27.1)	82 (27.2)	160 (26.6)	
		Never	43 (32.8)		55 (32.4)	97 (32.2)	195 (32.4)	
**Used smoking cessation apps before?**								1.4 (.48)
		Yes	77 (58.8)		93 (54.7)	158 (52.5)	328 (54.5)	
		No	54 (41.2)		77 (45.3)	143 (47.5)	274 (45.5)	
**Made quitting attempt using an app? (n=328)**								1.0 (.60)
		Yes	56 (46.2)		74 (79.5)	118 (74.6)	248 (75.6)	
		No	21 (53)		19 (20)	40 (25)	80 (24)	

**Table 2:** Number of participants who have used any type of smoking cessation apps in the past.

		Made quitting attempt		
Type of smoking cessation app	Used by number of participants^a^	Yes n (%)	No n (%)	χ^2^ (*P*)
Smoking counters (eg, count smoke free days or number of smoked cigarattes)	174	143 (82.2)	31 (17.8)	7.9 (.005)
Motivation (provide motivational messages)	113	86 (76.1)	27 (23.9)	0.0 (.99)
Text information (provide text info about smoking cessation)	76	59 (77)	17 (22)	0.1 (.75)
Combination of different types	68	46 (67)	22 (32)	2.4 (.12)
Hypnosis	51	42 (82)	9 (17)	1.0 (.29)

^a^Some participants have used more than one app.

### Participants' Use of Other Health Related Apps

Half of our respondents 50.2% (302/602) had used other health related apps in the past. Of these, 89.4% (270/302) were using health related apps at least weekly and 21.2% (64/302) daily. Participants were asked–“Have you ever checked the credibility of the developer or publisher of the health apps that you are currently using?”, and we found that 77.5% (234/302) of them had never checked and there was no difference by country (χ^2^=0.3, *P*=.86). Of those who had used health related apps in the past, 51.9% (157/302) used “Diet and Weight Management” apps, followed by 36.4% (110/302) “Training and Physical Activity,” 31.7% (96/302) “Health and Medication Information,” 23.2% (70/302) “Pregnancy and Ovulation Calculators and Calendars,” and 19.5% (59/302) “Medication Intake Reminder.” Very few participants had used “Diabetes Management” and “Asthma Control or Management” apps, 4.3% (13/302) and 3.3% (10/302) respectively.

### Information Ratings

Few app users rated the information, with most rating only some sections. “Smoking Consequences” information received ratings from 105 participants; with 75.2% (79/105) agreeing it had motivated them to quit. “Quitting Assistance Options” received 93 ratings; with 54% (51/93) agreeing it motivated them to quit, and 8% (8/93) disagreeing. “Myth Busting” also received 93 ratings, 64% (60/93) agreeing and 10% (10/93) disagreeing. “Before Quitting” received 87 ratings, with 70% (61/87) agreeing, “After Quitting” received 85, with 89% (76/85) agreeing. “After Quitting” and “Smoking Consequences” received the most positive responses, while “Quitting Assistance Options” received the least. However, the average number of times participants looked at any information category was 5.4, with no significant differences by country (χ^2^, *P*=.85). Chi-square analysis revealed a significant difference in stage of change between participants who looked at the five information categories, and those who did not (*P*=.03). Further logistic regression analysis controlling for age, gender, education, country, and time of recruitment showed that being in the “Preparation stage” or “Willing to quit in the next 30 days” were associated with looking at all the information (OR 1.7, 95% CI 1.1-2.7).

## Discussion

### Results Summary

In this study, 1638 participants from Australia, the United Kingdom, and the United States downloaded a free smoking cessation app over a 1-year period, and 36.75% (602/1638) of them completed an in-app questionnaire. The majority of respondents 77.2% (465/602) were willing to quit within the next 30 days, and 67.6% (407/602) have tried to quit at least once in the past year. Almost half had used smoking cessation apps in the past, and most had never checked the credibility of their health apps’ publisher. Most respondents never sought “Quitline” help 88.7% (401/452), or health care professional help 71.7% (324/452) in the last 12 months. Those who tried to quit twice or more in the last year, and those who find it difficult to refrain from smoking in banned areas, were more likely users of smoking cessation apps in the past.

### Number of App Downloads

In this study, we have examined the app uptake naturally without external promotion, and this may explain the small number of downloads over one year. In addition, the fact that smoking prevalence in the included countries varies from 15% to 20% with the lowest in Australia 15% [22] and highest in the United Kingdom 20% [23]. Therefore, the app downloads might be affected by the country population, prevalence of the condition, and the smartphone uptake in each country. Moreover, apps can also be affected by the app store rankings (where the apps are ranked higher), resulting in more exposure, and consequently more downloads [24].

Although the ownership of Android OS devices is twice that of Apple devices, the uptake of this study app was more than 2 times less in the Android Market over the 12-month period. Another study reported a low uptake of Android apps in the United States, where only 45 participants downloaded the study app during 2 months [25]. Interestingly, the study reported that more than 100 email messages and phone calls were received from Apple users showing interest in downloading the app whenever it was available in the Apple App Store [25]. Thus, even though Android OS users are double the Apple OS users, due to the fact that some Android devices are very inexpensive compared to Apple devices, we assume that some Android users are not recognizing it as a smartphone and are therefore not interested in apps, or maybe they do recognize it, but just do not have a need for the apps.

A number of users from other countries have been able to download the app at 6.8% (32/473) in the Android Market and 5.56% (71/1278) in the Apple store. Future studies should not take the app store function of limiting the app to a specific country for granted, and they may have to implement extra functions in the app to validate if the users are actually from the countries of interest, for example, a location identification service to know the users’ country the first time they open the app (as was used in this study). The ability of users from countries outside of this study to download this study app might be due to their using app store accounts registered for one of the countries of interest, or some other users might be using a proxy Internet connection that uses an IP of one of the countries of interest in this study.

This study app was able to reach smokers in the countries included in this study. Participants were similar in age and number of previous quitting attempts, and most sought smoking cessation help only in this new medium and not from professionals. This might be due to the app’s easier accessibility, privacy, anonymity, and portability. However, the documented low quality of smoking cessation apps, exaggerated claims of effectiveness, and the large number of prosmoking apps that claim smoking cessation, all help decrease these apps’ usefulness and lead to failed quitting attempts. Although there were almost 75.6% (248/328) of participants who had tried smoking cessation apps in the past to quit for good, only 33.0% (82/248) abstained for more than one week, which might reflect the available apps’ low quality.

### Number of Smoking Cessation Attempts

Previous quit attempts were associated with a two-fold increase in the use of smoking tracking cessation apps. Investigators have not yet explored the reasons for this. However, it might be due to design, popularity, or smartphones’ constant proximity, letting users monitor their progress anywhere and anytime [26], reinforcing tracking’s already documented effectiveness [27-29].

A three-fold increase in smoking cessation app use was associated with three or more quit attempts in the previous year. This might be due to the documented positive relationship between motivation to quit and number of quitting attempts [30,31], and the number of previous quitting attempts as an independent predictor of making a new quitting attempt [32]. In addition, respondents who found difficulty in refraining from smoking in banned areas were approximately twice as likely to try smoking cessation apps. This finding is consistent with other studies that found that smokers who lived or worked under a smoking ban were more likely to report quitting attempts [33,34].

### Previous Health Apps Use

There were 50.2% (302/602) of participants that used health related apps in the past, and of those, about 89.4% (270/302) used them at least weekly, but 77.5% (234/302) never checked the credibility of the health app publisher. Although we have not provided a specific definition of app “credibility” and relied on the users’ self-definition, this still poses a problem since many studies indicate low reliability and quality in all health related apps covering such topics as smoking [1,16], asthma [35], cancer [36], and pain management [37]. Thus, there is a need for better, evidence-based health apps, and more information about the quality of the current ones. App stores might also cooperate with public health institutions and researchers to improve the quality of health related apps and their reach.

### Response Rate

The feasibility of using smartphones as a research tool shows some promise with an unprompted response rate of 36.75% (602/1638) to our in-app questionnaire, and response rates as high as 44.9% (131/292) in Australia, and as low as 33.3% (170/511) in the United Kingdom. However, the response rate for rating the page content was very low and may be due to the fact that the page rating did not provide the users with feedback as was done in the questionnaire. A recent study explored using personalized feedback as incentive to increase compliance in Web-based questionnaires [38]. In addition, the variation in the rating responses may be due to the design of the rating process, as we have included the rating of each information page at the end of it. Asking the participants to rate all the pages at once after reading them all at once may eliminate the response variation problem.

### Study Limitations

This study also examined the feasibility of a new research recruitment methodology. There are no data available to adjust the nonrespondents or self-selection bias for such a study. Moreover, one of the limitations of this study cross-sectional methodology is the inability to identify the nonrespondents characteristics. Although the sampling method of this exploratory feasibility study was limited by selection bias, it provides for the first time to our knowledge, some useful data to suggest that smoking cessation apps are being used by people who want to quit and may also be a feasible tool for smoking cessation support and evaluation. Future studies could increase the response rate by implementing a reminder function in the app or use the push-notification services.

### Conclusions

This exploratory feasibility study shows that smartphone apps are a promising medium to reach smokers in Australia, the United Kingdom, and the United States. The countries differed little in some demographics and smoking status. Current smokers from the three countries, mostly ready to quit in the near future, but eschewing professional help, have downloaded smoking cessation apps in the past and tried using them to quit; however, the low quality of apps in the field undermines these efforts. Thus, this study has shown that smartphone smoking cessation apps can reach smokers across multiple nations. This paper has also shed some light on participants’ use of other health related apps and identified an alarming trend of consumers using health apps without knowing the credibility of its publishers.

## Abbreviations

apps: applications
IP: Internet Protocol
OS: operating system
QA: Quit Advisor

## References

[1] BinDhim NF, Freeman B, Trevena L (2014). Pro-smoking apps for smartphones: The latest vehicle for the tobacco industry?. Tob Control.

[2] Mackay M (2012). Mobile Industry Group.

[3] (2012). Ofcom.

[4] (2012). Nielsenwire.

[5] Arthur C The Guardian.

[6] Pope S (2009). Apple Press Info.

[7] Sims D (2013). Apple Press Info.

[8] Pope S (2011). Apple Press Info.

[9] (2012). Android official blog.

[10] (2011). The official Google blog.

[11] Stoddard J, Delucchi K, Muñoz R, Collins N, Stable EP, Augustson E, Lenert L (2005). Smoking cessation research via the internet: A feasibility study. J Health Commun.

[12] Lenert L, Muñoz RF, Stoddard J, Delucchi K, Bansod A, Skoczen S, Pérez-Stable EJ (2003). Design and pilot evaluation of an internet smoking cessation program. J Am Med Inform Assoc.

[13] Etter JF, Perneger TV (2001). Effectiveness of a computer-tailored smoking cessation program: A randomized trial. Arch Intern Med.

[14] An LC, Klatt C, Perry CL, Lein EB, Hennrikus DJ, Pallonen UE, Bliss RL, Lando HA, Farley DM, Ahluwalia JS, Ehlinger EP (2008). The RealU online cessation intervention for college smokers: A randomized controlled trial. Prev Med.

[15] Bock B, Graham A, Sciamanna C, Krishnamoorthy J, Whiteley J, Carmona-Barros R, Niaura R, Abrams D (2004). Smoking cessation treatment on the Internet: Content, quality, and usability. Nicotine Tob Res.

[16] Abroms LC, Padmanabhan N, Thaweethai L, Phillips T (2011). iPhone apps for smoking cessation: A content analysis. Am J Prev Med.

[17] Valdivieso-López E, Flores-Mateo G, Molina-Gómez JD, Rey-Reñones C, Barrera Uriarte ML, Duch J, Valverde A (2013). Efficacy of a mobile application for smoking cessation in young people: Study protocol for a clustered, randomized trial. BMC Public Health.

[18] BinDhim NF, McGeechan K, Trevena L International clinical trial registry platform.

[19] Heatherton TF, Kozlowski LT, Frecker RC, Fagerström KO (1991). The Fagerström test for nicotine dependence: A revision of the Fagerström Tolerance Questionnaire. Br J Addict.

[20] DiClemente CC, Prochaska JO, Fairhurst SK, Velicer WF, Velasquez MM, Rossi JS (1991). The process of smoking cessation: An analysis of precontemplation, contemplation, and preparation stages of change. J Consult Clin Psychol.

[21] Velicer WF, Fava JL, Prochaska JO, Abrams DB, Emmons KM, Pierce JP (1995). Distribution of smokers by stage in three representative samples. Prev Med.

[22] Australian Institute of Health and Welfare (2011). Drug statistics series no. 25 Cat. no. PHE 145.

[23] (2013). Action on Smoking and Health.

[24] BinDhim NF, Freeman B, Trevena L (2013). Pro-smoking apps: Where, how, and who are most at risk. Tob Control.

[25] Peck JL, Stanton M, Reynolds GE (2014). Smartphone preventive health care: Parental use of an immunization reminder system. J Pediatr Health Care.

[26] Piasecki TM, Richardson AE, Smith SM (2007). Self-monitored motives for smoking among college students. Psychol Addict Behav.

[27] McFall RM (1970). Effects of self-monitoring on normal smoking behavior. J Consult Clin Psychol.

[28] McFall RM, Hammen CL (1971). Motivation, structure, and self-monitoring: Role of nonspecific factors in smoking reduction. J Consult Clin Psychol.

[29] Febbraro GA, Clum GA (1998). Meta-analytic investigation of the effectiveness of self-regulatory components in the treatment of adult problem behaviors. Clin Psychol Rev.

[30] Gigliotti A, Laranjeira R (2005). Habits, attitudes, and beliefs of smokers in four Brazilian capitals. Rev Bras Psiquiatr.

[31] Rose JS, Chassin L, Presson CC, Sherman SJ (1996). Prospective predictors of quit attempts and smoking cessation in young adults. Health Psychol.

[32] Li L, Feng G, Jiang Y, Yong HH, Borland R, Fong GT (2011). Prospective predictors of quitting behaviours among adult smokers in six cities in China: Findings from the International Tobacco Control (ITC) China Survey. Addiction.

[33] Farkas AJ, Gilpin EA, Distefan JM, Pierce JP (1999). The effects of household and workplace smoking restrictions on quitting behaviours. Tob Control.

[34] Glasgow RE, Cummings KM, Hyland A (1997). Relationship of worksite smoking policy to changes in employee tobacco use: Findings from COMMIT. Community Intervention Trial for Smoking Cessation. Tob Control.

[35] Huckvale K, Car M, Morrison C, Car J (2012). Apps for asthma self-management: A systematic assessment of content and tools. BMC Med.

[36] Pandey A, Hasan S, Dubey D, Sarangi S (2013). Smartphone apps as a source of cancer information: Changing trends in health information-seeking behavior. J Cancer Educ.

[37] Rosser BA, Eccleston C (2011). Smartphone applications for pain management. J Telemed Telecare.

[38] Bälter O, Fondell E, Bälter K (2012). Feedback in web-based questionnaires as incentive to increase compliance in studies on lifestyle factors. Public Health Nutr.

